# Inhibitors of Serine Proteases in Regulating the Production and Function of Neutrophil Extracellular Traps

**DOI:** 10.3389/fimmu.2016.00261

**Published:** 2016-06-30

**Authors:** Pawel Majewski, Monika Majchrzak-Gorecka, Beata Grygier, Joanna Skrzeczynska-Moncznik, Oktawia Osiecka, Joanna Cichy

**Affiliations:** ^1^Department of Immunology, Faculty of Biochemistry, Biophysics and Biotechnology, Jagiellonian University, Krakow, Poland

**Keywords:** neutrophil extracellular traps, neutrophil elastase, SLPI, serpin B1, plasmacytoid dendritic cells, psoriasis

## Abstract

Neutrophil extracellular traps (NETs), DNA webs released into the extracellular environment by activated neutrophils, are thought to play a key role in the entrapment and eradication of microbes. However, NETs are highly cytotoxic and a likely source of autoantigens, suggesting that NET release is tightly regulated. NET formation involves the activity of neutrophil elastase (NE), which cleaves histones, leading to chromatin decondensation. We and others have recently demonstrated that inhibitors of NE, such as secretory leukocyte protease inhibitor (SLPI) and SerpinB1, restrict NET production *in vitro* and *in vivo*. SLPI was also identified as a NET component in the lesional skin of patients suffering from the autoinflammatory skin disease psoriasis. SLPI-competent NET-like structures (a mixture of SLPI with neutrophil DNA and NE) stimulated the synthesis of interferon type I (IFNI) in plasmacytoid dendritic cells (pDCs) *in vitro*. pDCs uniquely respond to viral or microbial DNA/RNA but also to nucleic acids of “self” origin with the production of IFNI. Although IFNIs are critical in activating the antiviral/antimicrobial functions of many cells, IFNIs also play a role in inducing autoimmunity. Thus, NETs decorated by SLPI may regulate skin immunity through enhancing IFNI production in pDCs. Here, we review key aspects of how SLPI and SerpinB1 can control NET production and immunogenic function.

## Neutrophil Serine Proteases in Neutrophil Biology

Neutrophils, key immune cells for protection against microbial infection, are also associated with a range of pathologies, including autoinflammatory diseases, such as systemic lupus erythematosus (SLE) and psoriasis (1–3). Neutrophils are a rich source of proteolytic enzymes, including serine proteases. The enzymatic activity of serine proteases depends on a catalytic triad that contains a serine residue. Four active serine proteases, neutrophil elastase (NE), cathepsin G (CatG), proteinase 3 (PR3), and neutrophil serine protease 4 (NSP4), as well as azurocidin, an enzymatically inactive serine protease homolog, were characterized in neutrophils (4, 5). Serine proteases are synthesized early in granulocyte development, during the promyelocytic stage of granulopoiesis in the bone marrow, and require N-terminal trimming by dipeptydyl peptidase I (DPPI) for activation (6–8). Under homeostatic conditions, the proteases are stored in a catalytically active form in the azurophilic granules of circulating granulocytes (4). Neutrophils are equipped with heterogeneous granules, which are classified into four subsets: primary or azurophilic granules, formed first during granulopoiesis and containing myeloperoxidase (MPO) and the serine proteases as their hallmark proteins; secondary or specific granules, containing lactoferrin and cathelicidin; tertiary or gelatinase granules, with gelatinase and lysozyme; and finally, secretory granules, with complement and chemotaxis receptors (4, 9). As pre-stored agents, neutrophil serine proteases can be quickly engaged to provide protection against microbial challenge, either by degrading internalized microbes or upon release from activated neutrophils. The serine proteases are important contributors to the physiological response to infection, both as antimicrobial agents and as immunomodulators. Although serine proteases, such as NE and CatG, can kill microbes by virtue of their antimicrobial activity unrelated to their digestive potential (10), these enzymes can also restrain microbial growth through the processing of microbial and host proteins. For example, they cleave virulence factors of enterobacteria (11) or liberate host antimicrobial peptides from their inert precursor proteins. The latter mechanism was reported for human cathelicidin hCAP-18, which is cleaved into the potent antimicrobial peptide LL37 by PR3 (12). Serine proteases also participate in a defense against microbes through limiting microbial spreading. The underlying mechanism involves degradation of an inhibitor of coagulation, tissue factor pathway inhibitor (TFPI) by NE, thereby fostering production of intravascular fibrin barriers that sequester bacteria (13).

The immunomodulatory function of neutrophil serine proteases depends to a large extent on the regulation of the bioavailability of adhesion molecules, cell surface receptors, growth factors, cytokines, and chemoattractants (4). For example, several cytokines belonging to the IL1 superfamily, such as IL1β, IL18, and IL33, have been reported to be processed into biologically active forms by NE, CatG, and/or PR3 (14–16). Given the crucial role of these cytokines in inflammatory responses to infection or sterile tissue damage, processing of these cytokines alone by neutrophil serine proteases may have far-reaching consequences for a number of the host defensive strategies. Likewise, by triggering the chemotactic activity of the inert chemoattractant proteins, such as chemerin (17, 18), or increasing the chemotactic potential of chemokines, such as CXCL8 (19), neutrophil serine proteases may mobilize specific immune cells to sites of inflammation. On the other hand, the NE-mediated proteolytic degradation of the chemokine CXCL12 and its receptor CXCR4, which disrupts the CXCL12/CXCR4 chemotactic pathway in the bone marrow, facilitates the mobilization of hematopoietic stem cell precursors from the bone marrow into the circulation in response to mobilizing agents, such as G-CSF (20). Together, these findings indicate that by activating or deactivating cell-guiding molecules, the serine proteases provide an important layer of control over cell recruitment.

The enzymes also influence neutrophil development and the functional state of the cell, including apoptosis and the formation of neutrophil extracellular traps (NETs). Mutations in the gene encoding NE-*ELANE* are a leading cause of severe congenital neutropenia (SCN), a disorder leading to a lack of mature neutrophils (21, 22). However, pathogenic *ELANE* mutations are distributed throughout NE, and at least some *ELANE* mutants retain NE activity (23), indicating that neutropenia is not a result of impaired NE proteolytic function. Indeed, recent advances suggest that the pathogenesis of *ELANE* mutations is associated with NE mislocalization, the accumulation of NE in the ER and other cytosolic regions outside of the azurophilic granules, and the activation of the unfolded protein response/ER stress. These alterations in turn lead to the death and differentiation arrest of granulocytic precursors (promyelocytes). Notably, the sequestration of mutated NE in azurophilic granules of myeloid precursor cells, as well as neutrophil maturation, can be rescued by a small, cell-permeable NE inhibitor, sivelestat, given in combination with low-dose G-CSF (23). Although sivelestat may also affect cellular responses in a manner independent of NE inhibition (24), these findings suggest that a NE inhibitor protects differentiating granulocytes against the activity of the mislocalized NE and that the impaired intracellular trafficking of NE can be corrected in the presence of a NE inhibitor.

Neutrophils have a short life span relative to other cells and are subjected to caspase-3-mediated spontaneous death, which phenotypically fits the profile of apoptotic cell death (25). Apoptosis is triggered and executed *via* intracellular cysteine proteases-caspases. The main effector protease that drives the terminal stages of cell death is caspase-3. This protein requires proteolytic cleavage for apoptotic activity. Among the key activatory enzymes are caspase-9 and caspase-8. It was recently reported that although cleavage of caspase-3 was integral to the death of aging neutrophils, it was independent of the proteolytic activity of caspase-8 or caspase-9. Instead, PR3 leaking from azurophilic granules into the cytosol was found to regulate caspase-3 activation and cell death in aging neutrophils (25).

Similar to apoptosis, neutrophil serine proteases have been shown to contribute to the formation of NETs. This process, called NETosis, is associated with irreversible cell state changes, but in a manner distinct from apoptotic death (26).

## Role of Serine Proteases in NET Formation

Neutrophil extracellular traps are web-like DNA structures extruded into the extracellular environment by activated neutrophils. A wide range of stimuli triggers NETosis, including Gram-positive and Gram-negative bacteria, such as *Staphylococcus aureus* (26, 27) and *Shigella flexneri* (28); the fungus *Candida albicans* (29); parasites, such as *Leishmania amazonensis* (30); and viruses, such as HIV-1 (31). NET formation is also induced by host-derived inflammatory mediators, such as hydrogen peroxide (H_2_O_2_) (26), the cytokines, such as IL17 and TNFα (32), chemokines, such as CXCL8 (28), monosodium urate (MSU) (33), cholesterol (34) or calcium carbonate crystals (35), antibodies (36), or antibody–antigen complexes (2). Synthetic chemicals, such as phorbol ester (PMA) or ionophores, are the most potent inducers of NETosis commonly used in experimental systems.

Neutrophil extracellular traps are thought to represent a unique defense strategy against microbial infection. NET microbicidal function is aided by antibacterial proteins and peptides that are complexed with decondensed chromatin and mitochondria-derived DNA. These proteins include histones, which account for ~70% of all detected proteins in PMA-stimulated neutrophils, as well as serine proteases. Notably, NE is the most abundant non-histone NET-protein, accounting for ~5% of the total protein (29). In agreement with the high levels of NE in NETs revealed by proteomic analysis, a dominant role was also found for the NE-mediated proteolytic signature in NETs based on a functional activity assay (37). Whereas the major proteolytic activity associated with NETs derived from PMA-stimulated neutrophils was attributed to NE (~70%), all remaining neutrophil serine proteases (CatG, PR3, and NSP4), most notably CatG, contributed to the cleavage sites that were profiled in NET samples (37). These data suggest the proteolytic involvement of all neutrophil serine proteases in NET formation and/or function.

The mechanisms that underlie NET release are not yet fully characterized but are proposed to involve at least two strategies. One pathway can be triggered by specific microbes or PMA, takes 2–4 h to culminate in NET release, and is associated with plasma membrane perforation and neutrophil cell death (26). After activation, neutrophils undergo step-wise morphological changes, including chromatin decondensation, nuclear expansion, and nuclear envelope disintegration, which is followed by the intracellular assembly of nuclear and cytoplasmic components. Finally, DNA coated with nuclear and cytoplasmic proteins is deposited into the environment following plasma cell rupture and cell lysis (26). In the other pathway, neutrophils undergoing NET formation can extrude a fraction or their entire nuclear DNA mainly through nuclear budding and vesicular release. During this process, neutrophils can maintain their integrity and live cell function, such as migration and phagocytosis, at least for a couple of minutes when tested *in vivo* (38). This form of non-lytic NET extrusion, also known as vital NETosis, is triggered by complement-opsonized targets and occurs much more rapidly (<30 min). Since NET release that involves cell lysis is a slow process, potentially allowing microbes to exploit the open time window for infection, the rapid NETosis might prove particularly beneficial against infection (38).

However, rapid NET release by neutrophils was also reported in response to collagen-activated platelets as well as danger signals, such as MSU crystals (13, 33, 39), suggesting that rapid NETosis is not limited to microbes. Platelet and neutrophil dialog through NETs supports blood clotting (13, 40). NETs that are formed within the vasculature capture anticoagulants, such as TFPI, enabling proteolytic inactivation of TFPI on NETs by NE. As a consequence of TFPI degradation, fibrin formation is rapidly enhanced (13). NETs are not only induced by activated platelets but can also serve as a scaffold to platelets aggregation and red blood cells adhesion, thereby accelerating coagulation (40). On the other hand, MSU crystal deposits in joints can induce gouty arthritis. These MSU crystals required 10 min to trigger NETosis and NET aggregation in human neutrophils (33). Aggregated NET structures were formed when neutrophils at high densities were stimulated with the crystals, mimicking dense neutrophil infiltrates in the synovial fluid of individuals with gouty arthritis. The aggregation of NETs was found to be beneficial in the setting of inflammatory arthritis, since it promoted degradation of chemokines and cytokines, such as IL1β, that were entrapped in NETs, providing a potential mechanism for resolution of inflammation (33). Thus, rapid NETosis may potentially serve multiple functions.

One of the best characterized models of NET formation is based on the activation of neutrophils purified from human blood by PMA. The model depends on the production of reactive oxygen species (ROS) by the NADPH oxidase complex and involves NE as one of the major contributors to chromatin decondensation (41, 42).

Several lines of evidence support the critical involvement of NE in NET generation. First, inhibitors of NE proteolytic activity, such as small β-lactam-based, cell-permeable NE inhibitors, blocked chromatin decondensation and NET release in neutrophils derived from healthy volunteers (41). Moreover, neutrophils isolated from patients suffering from Papillon–Lefevre syndrome (PLS) failed to release NETs or were severely impaired in NET formation (43, 44). PLS is a disorder caused by loss-of-function mutations in the gene encoding DPPI, resulting in marked defects in the activities of serine proteases, including NE (43). Finally, NETs were not detected in the lungs of mice deficient in NE in a pulmonary model of *Klebsiella pneumoniae* infection (41) or in mice double-deficient in NE and PR3 in an experimental model of atherosclerosis (34). Although mouse neutrophils are much less prone to NET formation than human granulocytes, together, these data indicate that the genetic or functional deficiency of NE severely inhibits NETosis. However, the lack of NE did not prevent NET generation in an experimental model of deep vein thrombosis (24). These data suggest that NE, although linked to NETosis, is not a causative agent in this process, or that neutrophils do not exclusively rely on NE for NET formation. Indeed, chromatin decondensation, a critical step in NETosis, was reported to also be mediated by peptidylarginine deiminase 4 (PAD4). Whereas NE destabilizes chromatin structure *via* the processing of specific histones (41), PAD4 mediates chromatin decondensation through converting histone tail arginine residues to citrullines (45). PAD4 is the main PAD isozyme expressed in neutrophils out of five PADs present in human cells. Moreover, histone citrullination is catalyzed primarily by PAD4, whereas other PADs citrullinate multiple substrates out of the nucleus (46). These data suggest that chromatin decondensation and subsequent NETosis relies mainly on PAD4.

Although the relative contribution of NE and PAD4 to histone modification and the alteration of chromatin structure in NETosis remain to be determined, it is likely that both enzymes act as co-regulators or separate regulators of chromatin decondensation, depending on the type of NET stimulus. For example, PAD4 requires calcium for its enzymatic activity (47), and NETosis triggered by calcium influx is associated with the presence of citrullinated histones, such as citrullinated histone H3, in the activated neutrophils (48, 49). However, in contrast to the calcium ionophore-stimulated granulocytes, citrullinated histone H3 was hardly observed in neutrophils triggered to form NETs by PMA, suggesting that PAD4 is less required for the PMA-regulated NETosis pathway (24, 42).

As mentioned earlier, NE is confined to azurophilic granules in resting neutrophils. However, upon neutrophil activation, NE can translocate to the nucleus and aid in chromatin decondensation *via* core histone cleavage (41). NE translocation from the primary granules to the nucleus is dependent on ROS generated by NADPH oxidase and MPO, which assists in releasing active NE from the granules into the cytosol (50). According to the recently proposed model of NE translocation, NE in azurophilic granules is associated with several other granule proteins, including MPO, PR3, CatG, azurocidin, eosinophil cationic protein, defensin-1, lysozyme, and lactoferrin. This association is supported by the immunoprecipitation of these proteins with anti-NE antibodies from isolated and detergent-solubilized azurophilic granules. H_2_O_2_, the secondary product of NADPH oxidase, triggers the dissociation of the NE protein complex from intact azurophilic granules, releasing NE when the enzyme is still assembled with CatG and azurocidin. In the cytoplasm, NE binds to the actin cytoskeleton and possibly degrades F-actin to reach the nucleus (50). Notably, the accumulation of NE in the insoluble cytoskeleton fraction isolated from the activated neutrophils is facilitated in the presence of a small molecule inhibitor of NE. Because blocking of NE activity markedly reduces NE entry into the nucleus (41), together, these findings are consistent with the model in which active NE interacts with the cytoskeleton en route to the nucleus and the inhibition of NE activity arrests NE on the cytoskeleton, preventing NE from translocating to the nucleus (50). Defining F-actin as a potential cytoplasmic substrate for NE also indicates that this protease may regulate neutrophil migration *via* the disassembly of the actin cytoskeleton during NETosis. NE interference with actin dynamics is likely to disable cell movement and confine NETting neutrophils to the NET trigger site. This strategy, embraced by neutrophils undergoing PMA- or *C. albicans*-induced NETosis, differs considerably from the rapid, vital NETosis that coexists with the ability of neutrophils to crawl (38, 50). Although these differences imply that NE might not be involved in rapid NETosis, early NET release that occurred 10 min after neutrophil stimulation with *L. amazonensis* was reduced by a NE inhibitor but was not significantly affected by diphenyleneiodonium (DPI), which mainly inhibits NADPH oxidase-mediated ROS production (30). These data suggest that NE might also be involved in rapid, ROS-independent NETosis.

## Inhibitors of Serine Proteases in NET Generation

Small molecule, exogenous NE inhibitors suppressed NET formation, suggesting that endogenous inhibitors of serine proteases might regulate NETosis in similar fashion. Notably, neutrophils contain multiple serine protease inhibitors (51, 52), but the roles of SerpinB1 and secretory leukocyte protease inhibitor (SLPI) are the best known in the context of neutrophil function (52, 53).

### SerpinB1

SerpinB1, also known as leukocyte elastase inhibitor (LEI) or monocyte/NE inhibitor (MNEI), is a member of the serpin family of serine protease inhibitors. Serpins are proteins characterized by a unique tertiary structure that employs a suicide-substrate-like mechanism to deactivate their target proteases (54). The inhibitors expose their reactive site loop as a substrate for a cognate protease. The protease cleaves the loop, which leads to extensive conformational changes of the serpin, resulting in protease entrapment in a tight covalent complex (55). Human serpins are divided into nine clades, named from A to I (56). SerpinB1 is a 42-kDa protein and is a member of the clade B serpins. Among the neutrophil serine proteases, SerpinB1 inhibits NE, PR3, and CatG. Notably, SerpinB1 is one of the most efficient inhibitors of NE (57, 58). SerpinB1 is mainly expressed in macrophages and neutrophils and accumulates at high levels in the cytoplasm and granules of neutrophils (59). It lacks a signal peptide and is not secreted to the extracellular environment *via* the classic secretory pathway. However, the detection of SerpinB1 in extracellular localizations, possibly as a result of cell necrosis, was also reported (60, 61).

This multifunctional cytoplasmic protein acts as a protease inhibitor in its native form, but the cleavage of SerpinB1 by its cognate proteases can lead to the loss of antiprotease properties and gaining other functions, such as DNAse activity. A SerpinB1 derivative equipped with nuclease activity, called L-DNAse II, was isolated from porcine spleen (62). NE-mediated SerpinB1 conversion from an antiprotease to an endonuclease resulted from conformational alteration that exposed the endonuclease active site and a nuclear localization signal. The SerpinB1 derivative L-DNAse II was reported to have pro-apoptotic effects (63). The main features of SerpinB1 are summarized in Table 1.

**Table 1:** **The main characteristics of SerpinB1 and SLPI**.

	Main expression	Main localization	Main functions	Proteolytic targets in neutrophils
SerpinB1	Macrophages, neutrophils	Intracellular	Inhibitor of serine proteases, DNAse	NE, CatG, PR3
SLPI	Epithelial cells, neutrophils	Secreted	Inhibitor of serine proteases, Antimicrobial protein, Inhibitor of NFκB	NE, CatG

By contrast, data have been accumulating that SerpinB1 plays a pro-survival role in neutrophils. This role is exemplified by the recently reported regulation of the spontaneous death of aging neutrophils by SerpinB1, *via* counterbalancing the activity of PR3 during leakage of the protease from azurophilic granules (25). Whereas cytosolic SerpinB1 was found to form a complex with PR3 in neutrophils undergoing spontaneous death but not in freshly isolated neutrophils, the rate of spontaneous death was increased in neutrophils isolated from SerpinB1-deficient mice (25). These findings are consistent with the protective role of SerpinB1 against apoptosis.

An intrinsic defect in survival observed in neutrophils derived from SerpinB1 knock-out mice may also be caused by a higher propensity of these cells to die by NETosis. In a model of *Pseudomonas aeruginosa* lung infection, neutrophils infiltrating the lungs of SerpinB1-deficient mice exhibited excessive death. The cell death was accompanied by the presence of free NE, MPO, and DNA in the bronchoalveolar lavage fluid (BALF) (52). In *ex vivo* experiments, neutrophils devoid of SerpinB1 that were isolated from the BALF of the infected mice also generated more NETs than those from control mice. These findings demonstrated that NETosis was increased in SerpinB1-deficient mice in the setting of infection. Higher susceptibility of SerpinB1-deficient neutrophils to form NETs was also found when neutrophils isolated from the bone marrow of uninfected mice were subjected to treatment with native proinflammatory mediators, such as the chemokine CXCL2, or PMA, suggesting that SerpinB1 controls NETosis (52). The addition of recombinant SerpinB1, but not related serpins, to these *in vitro* activated neutrophils abrogated NET production (52). These findings indicated that SerpinB1 is a negative regulator of NETosis.

In response to PMA, SerpinB1-deficient mouse neutrophils demonstrated a higher tendency to expand their nuclei, indicative of chromatin decondensation. Moreover, in PMA-treated human neutrophils, SerpinB1, similar to NE, migrated to the nucleus and co-stained with NE and DNA on NETs formed by the stimulated cells. These results raised the possibility that SerpinB1 blocks NET formation *via* interfering with NE-mediated chromatin decondensation. However, SerpinB1 localization to the nucleus did not seem to involve forming a complex with NE. When NE was confined to the cytoplasmic region by neutrophil pretreatment with a chemical protease inhibitor, SerpinB1 could still translocate to the nucleus (52). Moreover, the enhanced NET formation observed in SerpinB1-deficient mice was not reversed by NE deletion (24). Therefore, NE might not be an exclusive SerpinB1 target in restricting NETosis. Given the multiplicity of its cognate proteases, it is also possible that, in the absence of NE, SerpinB1 might select other proteolytic targets to limit NET generation. As an alternative mechanism, nuclear SerpinB1 was proposed to interfere with PAD4 by blocking PAD4 access to histone tails (52). This role for SerpinB1 in regulating chromatin decondensation is supported by the ability of the inhibitor to tightly associate with condensed chromatin (64).

### Secretory Leukocyte Protease Inhibitor

Secretory leukocyte protease inhibitor, a single polypeptide cationic protein of 107 amino acids, is also known as antileukoprotease (ALP), bronchial secretory inhibitor (BI), human seminal inhibitor I (HUSI-I), cervix uterine secretion inhibitor (CUSI), mucous proteinase inhibitor (MPI), or secretory leukoprotease inhibitor (SLI) (65). The most well-documented role of SLPI is the inhibition of serine proteases, including human NE and CatG but not PR3 (66), Table 1. Beyond a role in neutralizing proteases, SLPI is also microbicidal and suppresses the activity of the transcription factor NFκB (67). SLPI is a canonical type of serine protease inhibitor, binding to proteases through the exposed binding loop, which in conformation is complementary to the enzyme’s active site (66). The inhibitor is composed of two four-disulfide core domains, also called whey acidic protein (WAP) domains. The N-terminal WAP I and C-terminal WAP II domains share 35% homology (68), but their biological function is distinct. The WAP II domain is primarily responsible for the antiprotease activity of the SLPI. The biological function of the N-terminal WAP I domain is less well understood, although the antimicrobial potential of SLPI is thought to mainly reside in this domain (67).

In contrast to SerpinB1, SLPI is predominantly secreted and found primarily at mucosal surfaces as a product of epithelial cells. The inhibitor is also expressed in leukocytes, including neutrophils, macrophages, and dendritic cells (67). Despite the presence of a signal peptide, indicative of cell secretion, SLPI has intracellular targets, suggesting that it might be retained in cells. However, the inhibitor can penetrate cellular membranes and potentially be acquired from adjacent cells. Such loading with SLPI, mimicked in experimental systems by cell treatment with the exogenous inhibitor, appears to be functionally relevant. For example, SLPI produced by epithelial cells lining tonsillar crypts restrains the production of antibodies in adjacent B cells (69).

Although SLPI was shown to inhibit a wide spectrum of proteases, one of its main actions is likely to be the inhibition of NE because the complex of SLPI with NE is the strongest among complexes of SLPI with any other proteases (66, 70). Notably, SLPI is thought to be the major inhibitor of NE present in the neutrophil cytosol (51). According to another report, SLPI is primarily stored in secondary granules in neutrophils (71). As SLPI, similar to NE, is likely to migrate between different cell compartments in response to neutrophil stimulation, the presence of SLPI in the cytosol or in specific granules might reflect different activation statuses of neutrophils. The main features of SLPI are summarized in Table 1.

Secretory leukocyte protease inhibitor plays a regulatory role in granulopoiesis (72) and, similar to SerpinB1, inhibits apoptosis in circulating neutrophils (73). The mechanism underlying its antiapoptotic activity remains to be determined. However, the protective role of SLPI in apoptosis might be reminiscent of SerpinB1 counteracting PR3, although its activity must be directed against other proteases because SLPI does not inhibit PR3.

Secretory leukocyte protease inhibitor may also serve to protect cells from the harmful effects of NETosis. This conclusion stems from the observation that stimulation of human neutrophils with PMA, TNFα, or *S. aureus* in the presence of exogenous SLPI, but not another native NE inhibitor, α1-proteinase inhibitor, substantially decreased NET release. Exogenous SLPI mainly localized to the cytoplasm of resting neutrophils, but upon stimulation it relocated to the nucleus and inhibited histone cleavage. Endogenous SLPI was also found to co-localize with NE in the nuclei of *in vitro* activated human neutrophils, or in neutrophils infiltrating the skin of patients with the autoinflammatory skin disease psoriasis (53). Together, these data suggested that the protective effect of SLPI in NET formation might result from constraining NE-mediated histone processing. Notably, although histone cleavage was efficiently blocked by added SLPI independently of the NET-triggering stimulus, in response to PMA, neutrophils appeared to degrade more histones than neutrophils stimulated with *S. aureus* (53). This observation further points to divergent mechanisms underlying NETosis, with *S. aureus* possibly relying more on other pathways. Moreover, the inhibition of NE activity was unlikely to be solely responsible for the SLPI tailoring effect on NETosis because SLPI mutants devoid of inhibitory activity against NE were still capable of restraining NET formation in stimulated neutrophils, albeit to a lower degree than the fully active SLPI (53). Given the inhibitory effects of SLPI against multiple proteolytic enzymes, one possible mechanism whereby SLPI may interfere with NETosis is to counteract other proteases. In another scenario, the independent antiprotease activity of SLPI might be involved in blocking NET release. In agreement with the anti-NET effect of SLPI, the inhibitor deficiency led to excessive NETosis in *in vitro* activated bone marrow neutrophils. The negative regulation of NET formation by SLPI was further supported by an *in vivo* psoriasis model. This model demonstrated that SLPI^−/−^ neutrophils infiltrating psoriatic skin formed twice as many NETs as WT neutrophils (53).

Because neutrophil treatment with exogenous SLPI resulted in a partial decrease in NET generation, other inhibitory parallel pathways must exist. The overlapping but distinct activities of SLPI and SerpinB1 against neutrophil proteases (Table 1), as well as the structural and functional heterogeneity between the inhibitors, suggest that SLPI and SerpinB1 may act in synergy to control NETosis.

## Inhibitors of Serine Proteases in NET Function

Uncontrolled NET production in chronic inflammatory states has serious consequences. For example, NET formation may contribute to the pathogenesis of autoimmune diseases, as NETs are cytolytic and are a potential source of autoantigens (32, 74, 75). Multiple NET components, including DNA, histones, MPO, PR3, LL37, CatG, and NE, are recognized by autoantibodies (76–78). The clinical measures of disease severity are often positively correlated with the titers of antibodies directed against NET components (79). As discussed earlier, SerpinB1 and SLPI may be a part of the defense system to cope with challenges imposed on the host by NET deposition. However, as regulators of NETosis, they may be externalized together with other NET components and impact immunity after extrusion from neutrophils. Although SLPI, as a secretory protein, is unlikely to be intrinsically immunogenic, intracellular SerpinB1, normally hidden in the cell, might be revealed as a consequence of NETosis and potentially provoke or enhance immune responses. Immunohistochemistry data indicated that *in vitro* activated human neutrophils release NETs with SerpinB1 and SLPI attached to DNA (52, 80). Likewise, SLPI was also found to decorate NETs in the affected skin of patients suffering from psoriasis (80, 81), suggesting that the presence of SLPI on NETs might be functionally relevant.

Psoriasis is a skin condition affecting on average 2–3% of the population all over the world (82). Most often, it manifests as erythematous cutaneous lesions covered with silvery scales. The disease is incurable and although it is not life threatening on its own, patients suffering from psoriasis exhibit a higher risk of developing comorbidities, such as other autoimmune disorders, metabolic syndrome, and cardiovascular disease (83, 84). On a cellular level, psoriasis is characterized by dysfunctional keratinocyte proliferation and differentiation, as well as inflammation elicited by abundant immune cells that are rare or absent in healthy skin (85). Among them are plasmacytoid dendritic cells (pDCs) and neutrophils. Although emerging data indicate that chronic inflammation in this disease is mediated primarily by Th17 cells and their signature product, IL17 (86), the pathological events underlying the initiation of the disease are much less understood (87). pDCs accumulate early in psoriatic skin lesions or pre-lesional skin adjacent to affected skin (85, 88), suggesting that pDCs are well-placed to contribute to early skin alterations. These cells are considered key producers of interferon type I (IFNI) (89). pDCs and IFNI were implicated in the pathogenesis of psoriasis and other autoimmune diseases, such as SLE (90, 91). The diseases exhibit broad activation of IFNI pathways (1). Direct evidence for a pathogenic role for pDC-derived IFNI in psoriasis is provided by a human/mouse skin xenograft model. In this model, the spontaneous conversion of the transplanted human uninvolved (normal appearing) skin of psoriasis donors into psoriatic skin lesions is prevented by blocking IFNI signaling or inhibiting pDC production of IFNI (92). As one of the key cell types involved in antiviral immunity, pDCs are well equipped with intracellular sensors of nucleic acids, such as TLR7 and TLR9, which recognize single-stranded RNA and DNA, respectively (89). However, pDCs can be activated in a TLR-dependent manner, not only by foreign but also by self RNA and DNA. In each case, pDC stimulation can lead to the production of IFNI and IFNI-driven immunity (93, 94). To be functional ligands for TLRs, host nucleic acids need to be of mitochondrial origin and/or form complexes with specific proteins, such as antimicrobial LL37 (93, 94) or NE together with SLPI (80). Notably, activated neutrophils are a likely source of these IFNI-triggering factors because they extrude both oxidized mitochondrial nucleoids (95, 96) and nuclear DNA decorated with the proteins (3). Mitochondria, along with the phagosome-localized NADPH oxidase complex, are major sites of ROS production, and mitochondrial DNA is highly susceptible to oxidation (97). This modification is required for the potent interferogenic potential of mitochondrial DNA (95). Although mitochondrial DNA can be extruded from cells without subsequent cell death and release of nuclear DNA (98), it is likely that neutrophils triggered by a suitable stimulus co-release oxidized mitochondrial and chromosomal DNA. This possibility is supported by recent findings that demonstrate that NETs are enriched in oxidized mitochondrial DNA when stimulated with ribonucleoprotein immune complexes (96). Therefore, unique structural or functional assets of host DNA, such as levels of oxidized mitochondrial DNA in the NET structure, and/or other NET DNA modifications, possibly resulting from the specific assembly of DNA with neutrophil proteins, may allow pDCs to recognize self DNA.

In psoriatic skin, induction of IFNI synthesis by neutrophil-derived self DNA in skin-infiltrating pDCs may depend on SLPI. Some insights into this came from the observation that NET-like structures, consisting of DNA, NE, or CatG, and SLPI were present in the affected skin of patients with psoriasis (80, 81). Moreover, pDCs were found to locate in close proximity to neutrophils and NETs (80), indicating that pDCs and SLPI-decorated NETs might be linked in controlling skin pathophysiology. Although not effective on its own, SLPI in complex with neutrophil DNA and its cognate enzymes, NE or CatG, induced a marked increase in the production of IFNI by pDCs (80, 81). This response was mediated by TLR9, suggesting that recognition of self DNA by intracellular TLR9 and/or activation of the receptor is facilitated by SLPI. Other inhibitors exposed on NETs can potentially also be involved in the stimulation of IFNI production in pDCs. Although the role of SerpinB1 in this process remains unknown, SLPI appears to have the selective capacity to induce the expression of IFNI in pDCs. In contrast to SLPI, neither the main plasma inhibitor of NE, α1-proteinase inhibitor, nor the main plasma inhibitor of CatG, α1-antichymotrypsin (99, 100), were effective at stimulating IFNI production by pDCs (80, 81). Therefore, regulation of the catalytic activity of serine proteases may not be a uniting or adequate property for an inhibitor to enable pDCs to produce IFNI. This possibility was further supported by the finding that SLPI lacking potent anti-NE activity was still equipped with pDC-stimulating functions. By contrast, the proimmunogenic ability of SLPI together with DNA and NE required enzymatically active NE because NE inhibited by a small synthetic inhibitor, or another inactive protein closely related to NE and embedded in NETs, azurocidin, did not stimulate pDCs to produce IFNI (80). The proimmunogenic properties of SLPI may primarily be related to its cationic nature because cationic peptides, such as LL37, display strong capacity to activate pDCs (101). However, cationicity alone does not appear to be sufficient for the stimulation of IFNI production in pDCs because the complex of SLPI and neutrophil DNA was unable to potently trigger IFNI production by pDCs (80).

**Figure 1:**
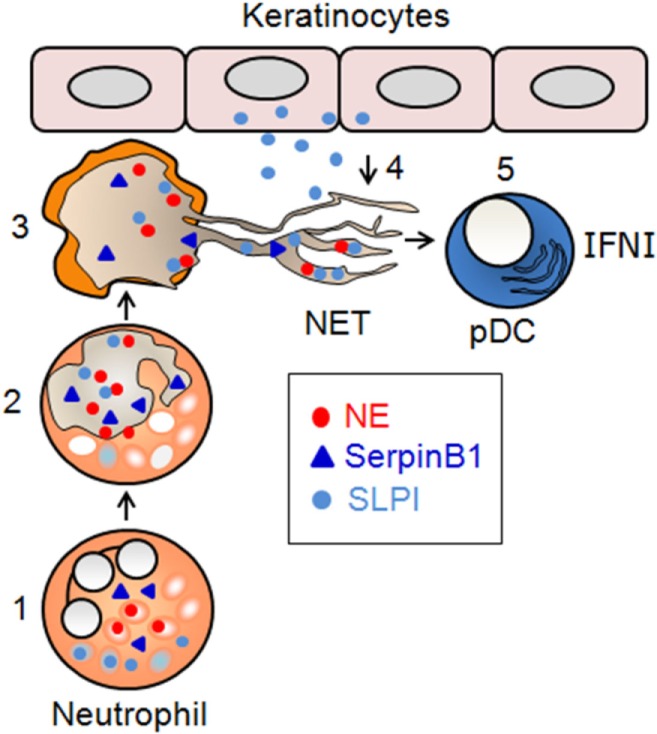
**Proposed involvement of SerpinB1 and SLPI in NET formation and immunogenic function**. (1) In resting neutrophils, NE localizes to primary granules, whereas SerpinB1 and SLPI localize to the cytoplasm and/or secondary granules. (2) In activated neutrophils that infiltrate psoriatic skin, NE translocates to the nucleus, where it contributes to chromatin decondensation. SLPI and SerpinB1 translocate independently to the nucleus, where they regulate NET formation at the level of chromatin decondensation. Once in the nucleus, SLPI restricts the NE-mediated cleavage of histones, whereas SerpinB1 limits chromatin decondensation through other, yet-to-be-identified mechanisms. (3) The inhibition of NET formation is partial, and the decondensed chromatin containing NE, as well as SerpinB1 and SLPI, is deposited into the extracellular milieu. (4) SLPI produced by keratinocytes in lesional psoriatic skin is sequestered on NETs. (5) SLPI-competent NETs stimulate the pro-inflammatory and/or skin healing function that results from skin damage through the production of IFNI by pDCs.

A role for SLPI in psoriasis was initially suggested by the observation that SLPI is markedly upregulated in the epidermis of psoriasis patients, particularly in keratinocytes (102). As described previously, neutrophils are a potential but not necessarily unique source of SLPI on NETs in psoriatic skin. Given the ability of SLPI to bind to DNA (103), it can be envisaged that SLPI produced by keratinocytes might dock to NETs deposited in psoriatic skin *via* skin-infiltrating neutrophils (Figure 1). In principle, NETs enriched in SLPI might be particularly suitable to prime pDC responses. NETs different in composition or protein levels were described earlier. Although the protein constitution in NETs might be influenced directly by the triggering stimulus (32), it is also likely that the stimulatory power of NETs may depend on the tissue context, by recruiting additional tissue-specific components to the externalized nuclear/mitochondrial nucleic acids.

The functional significance of SLPI on NETs in psoriasis remains to be determined. However, SLPI might be involved in several levels of NET regulation in inflamed skin, each potentially leading to different outcomes. As a NET component, SLPI is likely to have an impact on the production of IFNI by pDCs, facilitating IFNI-mediated immune and skin healing responses (67). The flipside is the generation of a potentially harmful stimulus (SLPI-decorated NETs) that can increase the risk of autoimmune inflammation. On the other hand, the ability of SLPI to inhibit NETosis in neutrophils makes it ideally suited for serving as a safeguard against the harmful effects of NETs. Either way, SLPI emerges as an important participant in innate immunity *via* the regulation of NET generation and immunogenic function (Figure 1).

## Author Contributions

All authors listed have made substantial, direct, and intellectual contribution to the work and approved it for publication.

## Conflict of Interest Statement

The authors declare that the research was conducted in the absence of any commercial or financial relationships that could be construed as a potential conflict of interest.

## Abbreviations

CatG, cathepsin G; DPPI, dipeptydyl peptidase I; IFNI, interferon type I; NE, neutrophil elastase; MPO, myeloperoxidase; MSU, monosodium urate; PAD4, peptidylarginine deiminase 4; pDCs, plasmacytoid dendritic cells; PMA, phorbol ester; PR3, proteinase 3; SLPI, secretory leukocyte protease inhibitor; TFPI, tissue factor pathway inhibitor.
